# Dispersive heterodyne cavity ring-down spectroscopy exploiting eigenmode frequencies for high-fidelity measurements

**DOI:** 10.1126/sciadv.adp8556

**Published:** 2025-01-29

**Authors:** Agata Cygan, Szymon Wójtewicz, Hubert Jóźwiak, Grzegorz Kowzan, Nikodem Stolarczyk, Katarzyna Bielska, Piotr Wcisło, Roman Ciuryło, Daniel Lisak

**Affiliations:** Institute of Physics, Faculty of Physics, Astronomy and Informatics, Nicolaus Copernicus University in Toruń, Grudziadzka 5, 87-100 Torun, Poland.

## Abstract

Measuring low light absorption with combined uncertainty <1 per mil (‰) is crucial for many applications. Popular cavity ring-down spectroscopy can provide ultrahigh precision, below 0.01‰, but its accuracy is often worse than 5‰ due to inaccuracies in light intensity measurements. To eliminate this problem, we exploit optical frequency information carried by the ring-down cavity electromagnetic field. Instead of measuring only the decaying light intensity, we perform heterodyne detection of ring-downs followed by Fourier analysis to provide exact frequencies of optical cavity modes and a high-fidelity dispersive spectrum of a gas sample from them. We demonstrate the sub–per-mil accuracy of our method, confirmed by the best ab initio results for CO line intensity and for HD line shape, and the long-term repeatability of our dispersion measurements at 10^−4^ level. We see potential for our approach in atmospheric remote sensing, isotope ratio metrology, thermometry, and establishment of primary gas standards.

## Teaser

Cavity ring-down spectroscopy enables substantially improved accuracy by measuring eigenmode frequencies of the optical cavity.

## INTRODUCTION

The challenge of measuring the shape and intensity of spectral lines with a relative accuracy of 10^−3^ and better is highlighted in numerous scientific, industrial, and metrological applications using sensitive optical spectroscopy. Regarding the effect of global warming, changes in the Earth’s climate are expected to affect the capacity of natural repositories of anthropogenic greenhouse gases (GHGs), which will generate a feedback response to climate change (*1*). To predict the evolution of these changes, the location of regional sources and sinks of GHG is essential. Spectroscopic retrieval models must exhibit sensitivity to changes in their concentration at the per-mil level, necessitating a laboratory spectrum accuracy of at least an equivalent magnitude (*2*). Any systematic errors are of great concern because they introduce regional bias that imitates sources and sinks of GHG. Particular attention is also required in measurements of the stable isotope ratio, as repeatability is compromised over time due to the aging of reference materials (*3*). A promising approach involves the spectroscopic measurement of the absolute isotope ratio from the ratio of the line intensities of these isotopes (*4*). This method has recently demonstrated (*5*) a relative combined measurement uncertainty at the sub–per-mil level, showing good agreement with the results obtained from other methods. However, to measure line intensities with such high accuracy, careful calibration of the light intensity detection system is necessary (*6*). Accurate measurements of the line intensity ratios are also the basis for the new concept of optical primary thermometry (*7*). With the current standard of using first principles to define the units of temperature, pressure, and number density (*8*), optical methods offer promising prospects for realizing primary gas standards (*9*). As molecules interact with electromagnetic radiation, the accurate measurements of the refractive index enable the determination of gas thermodynamic parameters. Cavity-based nitrogen refractometry with a relative precision of 10^−6^ holds the greatest potential for realizing an optical primary pressure standard (*10*, *11*) to date. On the other hand, individual spectral lines, shaped by molecular interactions, provide molecular selectivity for optical gas standards. Progress in the mutual development of line-shape theory and spectroscopic methods (*12*) motivates continuous improvement in ab initio accuracies of spectral line intensities (*13*, *14*), which opens new possibilities for developing gas mixture and pressure standards related to accurate measurement of line intensity.

Many of these applications use cavity ring-down spectroscopy (CRDS) (*15*) to quantitatively measure trace and weakly absorbing species in the gas phase. Traditional CRDS systems, widely used due to their simplicity, reliability, and calibration-free nature, with inherently high sensitivity and spectral resolution, have been improved with laser and cavity stabilization technologies (*16*, *17*) and optical frequency combs providing accurate absolute frequency axes (*18*). Although the best obtained relative precision exceeds 10^−5^, the determined line intensities can differ by up to several percent between spectrometers due to the limited ability to measure the undistorted ring-down signals (*6*, *19*). As long as the light is turned off quickly enough and the single mode is excited, the main factors that limit the accuracy of the vertical axis in CRDS are the nonlinearity of the amplitude and bandwidth of the detection system, which includes the detector itself, the hardware digitizing ring-downs, and electronic devices along the way. It was recently shown that digitizer nonlinearity can be reduced by the calibration to a metrology-grade reference digitizer (*6*). Alternatively, one can measure the dispersion spectrum of the sample obtained from central frequencies of optical cavity modes, the positions of which are shifted within the molecular resonance range (*20*, *21*). This calibration-free cavity mode dispersion spectroscopy (CMDS) uses only the dc part of the detection band, making it completely insensitive to the nonlinearity of the entire band. Moreover, CMDS is much less sensitive to the amplitude nonlinearity of the detector than traditional CRDS. However, due to the need for point-by-point scanning of each cavity mode profile, the CMDS is much slower than CRDS, which makes it more susceptible to various drifts. We note, however, that the CMDS speed problem can be solved, at the cost of much lower laser-to-cavity coupling efficiency due to the frequency mismatch, by using cavity buildup dispersion spectroscopy (CBDS) (*21*), the accuracy of which is similar to CMDS. Both approaches, CRDS and CMDS, discussed above, have recently shown the best results for the line intensity: sub–per-mil accuracy in measurements (*13*, *21*) and sub–per-mil agreement with ab initio results (*13*).

In this work, we exploit the optical frequency information carried by the ring-down cavity electromagnetic field, not used in conventional CRDS, for high-fidelity spectroscopic measurements. Instead of measuring only the decaying light intensity, we perform heterodyne detection of ring-downs followed by Fourier analysis not only to reduce noise on the ring-down signals (*22*–*24*) but mainly to provide exact frequencies of optical cavity modes and a dispersive spectrum of a gas sample from them. We demonstrate that our approach is insensitive to light intensity measurement inaccuracies that constitute a problem for most spectrometers using light intensity detection. Moreover, it allows the selection of the most linear range of a detector transfer function (TF), thus eliminating the major contribution to the measurement error in the traditional CRDS. We point out that, with a small change in configuration, any CRDS system using laser-cavity locking technology can be easily converted into a dispersive heterodyne CRDS (HCRDS) system, providing high accuracy. In other words, dispersive HCRDS combines the accuracy of CMDS (*21*) with the speed (*25*) and simplicity of conventional CRDS (*15*). Using the carbon monoxide (CO) line intensity as an example, we demonstrate the sub–per-mil accuracy of our method, confirmed by the best ab initio result (*13*), and the long-term repeatability of our dispersion measurements at 10^−4^ level. Per-mil accuracy of the hydrogen deuteride (HD) line intensity and per-mil consistency of the HD line intensity and shape with the ab initio results are achieved. The showcased high-accuracy examples offer promising insights into the potential application of our method in atmospheric remote sensing (*2*, *26*), isotope ratio metrology (*5*, *27*), and the establishment of primary gas standards (*9*) and thermometry (*28*).

## RESULTS

### The principle of heterodyne frequency detection of light decaying from an optical cavity mode

**Fig. 1.**
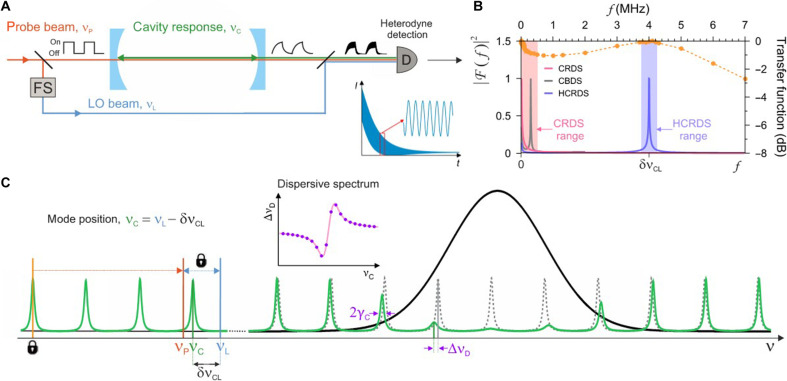
The principles of HCRDS. (**A**) The probe beam excites the optical cavity at arbitrary frequency $\nu_{P}$ close to the cavity resonant frequency, $\nu_{C}$. The cavity responds at the resonant frequency, $\nu_{C}$. The LO beam at frequency $\nu_{L}$ is frequency-shifted (FS) relative to the probe beam. After turning off the probe beam, it enables heterodyne detection of ring-down signals with the frequency ${\delta \nu }_{\text{CL}}=\nu_{L}-\nu_{C}$. (**B**) The power spectrum (PS) ${|ℱ\left(f\right)|}^{2}$ of the signals obtained by CRDS, CBDS, and HCRDS methods. The dots and the dashed line are the measured and fitted shape of the TF of the detection system used in CRDS and HCRDS measurements, respectively. The rectangles indicate the range of harmonic components of ${|ℱ\left(f\right)|}^{2}$ taken into account in the analysis of the CRDS and HCRDS signals. Fitting the Lorentz profile to the peak at ${\delta \nu }_{\text{CL}}$ provides the width and center of the cavity mode. (**C**) In the vicinity of the molecular resonance the cavity modes are shifted by dispersion and broadened by absorption. The probe beam frequency, $\nu_{P}$, is tuned with steps of FSR by the broadband EOM relative to the locking point, while the frequency difference $\nu_{L}-\nu_{P}$ is kept constant. For each spectral step $\nu_{C}$ is determined from the measured ${\delta \nu }_{\text{CL}}$. The frequency shifts $∆\nu_{D}\left(\nu_{C}\right)$ between cavity modes disturbed (continuous line) and undisturbed (dashed line) by the molecular line provide the HCRDS dispersion spectrum shown in the inset plot.

Immediate injection of probe light at the frequency $\nu_{P}$ into a high-finesse optical cavity begins the process of building a field inside the cavity. The field emerging the cavity is a finite sum of components oscillating at the input frequency $\nu_{P}$ with different phase shifts if the input field does not match the cavity mode. In the “Simulations” section, we showed that typical signal sampling conditions in the experiment make it possible, with a good approximation, to look at this phenomenon as a superposition of two fields. As soon as the laser field with frequency $\nu_{P}$ enters the cavity, the cavity begins to oppose it with its internal field centered around the cavity mode frequency $\nu_{C}$ (see Eq. 4 in the “Simulations” section). This cavity field decays exponentially, leading to damped oscillations with frequency $\nu_{C}-\nu_{P}$ at the cavity output until a steady state is reached in the cavity at frequency $\nu_{P}$ (*29*). If we now begin to turn off the probe laser at a rate of $\Gamma_{0}$, then the cavity resists it again. Damped oscillations with frequency $\nu_{C}-\nu_{P}$ appear again at the cavity output and continue until the laser field is turned off [see (*30*) and Eq. 7 in the “Simulations” section]. After this time, the remaining field leaving the cavity is the internal cavity field centered around the frequency $\nu_{C}$ and decaying exponentially at a rate $\Gamma_{C}$, if only $\Gamma_{0}\gg \Gamma_{C}$. The conventional CRDS detection system is insensitive to frequency measurement, which results in the incorrect assignment of the determined ring-down time constant, τ, to the laser frequency rather than the cavity resonant frequency. This frequency error depends on spectral widths of the laser and cavity mode. Detection of the light decay relative to a stable, local oscillator (LO) beam with frequency $\nu_{L}$ (Fig. 1A) allows one to extract missing information about the cavity resonance frequency and guarantees the correct frequency axis of the spectrum. The intensity emerging from the cavity, after beating with the LO beam, is

$$I_{\text{out}}\left(t\right)=I_{L}+I_{C}e^{-t/\tau }+2\eta \sqrt{I_{L}I_{C}}e^{-t/2\tau }\text{cos}\left(2\pi \;\delta \nu_{\text{CL}}t+\varphi_{L}\right)$$ (1)

where the first term is the LO intensity; the second is the exponential decay of the light from the cavity measured by conventional CRDS; and the third is the heterodyne beat, with frequency ${\delta \nu }_{\text{CL}}$, between the LO and cavity response fields. Here, η is the mode matching factor between the LO and decaying light beams and $\varphi_{L}$ is the phase shift between the LO and decaying fields introduced by the cavity and different optical path lengths. Further analysis of this signal, in the traditional sense of heterodyne detection, assumes using a band-pass filter to reduce low-frequency technical noise. Similar results provide an analysis of the high-frequency range of the Fourier power spectrum (PS) of the signal $I_{\text{out}}\left(t\right)$, shown in Fig. 1B. In addition, the PS analysis mitigates the potential heterodyne signal distortion that may arise in certain cases using electrical filters. The Lorentzian peak at frequency ${\delta \nu }_{\text{CL}}$ provides the position of the cavity mode $\nu_{C}=\nu_{L}-{\delta \nu }_{\text{CL}}$. Moreover, its full width gives the half-width of the cavity mode, $\gamma_{C}={\left(4\pi \;\tau \right)}^{-1}$. High precision of measurements of both quantities is guaranteed by the high stability of the LO frequency $\nu_{L}$ relative to the cavity resonances. We note that the Lorentzian peak at dc frequency does not provide additional information on molecular absorption for HCRDS presented here, but it could be used to estimate the LO frequency noise contribution to HCRDS. This low-frequency range of the PS, used by traditional CRDS, is usually affected by nonlinearities in the detection band. In addition, the PS of the buildup signal used by CBDS (*31*) may encounter the same problem. Hence, in CBDS, a compromise must be achieved between detuning the laser away from the cavity mode center toward higher beat frequencies and the resulting reduction in the beam power transmitted through the cavity. The HCRDS is insensitive to the detection nonlinearity problems (see the “Simulations” section). It allows one to select the optimal ${\delta \nu }_{\text{CL}}$ frequency so that the measurement of cavity mode parameters coincides with the most linear range of the detector’s bandwidth. Moreover, the symmetry of the Lorentz peak ensures that the determined cavity mode position is highly immune to nonlinearities in the light intensity measurement.

### Measurement of spectra using HCRDS

The idea of obtaining dispersive and absorptive spectra using the HCRDS method is presented in Fig. 1C. Precise measurement of the position and width of cavity modes requires tight locking of the continuous-wave laser to the cavity. In addition, to prevent thermal drift of the cavity modes comb, the cavity length is actively stabilized to another laser having long-term frequency stability of 11 Hz. The probe laser is split into two beams: one for exciting the cavity mode and the other, LO, serving as a reference for heterodyne detection of light decays. In the implemented approach, both beams are frequency-stepped using a broadband electro-optic modulator (B-EOM) and a microwave driver (*25*). Although such a configuration generates a series of sidebands on the laser, the optical cavity acts as a spectral filter, allowing only one of the excitation beam sidebands to resonate with the cavity. Similarly, in the case of heterodyne detection, the limited detection system bandwidth allows the decay beat signal to be observed with only one sideband of the reference beam. The ring-down decays are initiated after the excitation beam is turned off by an acousto-optic modulator (AOM) (see the “Experimental design” section). This AOM shifts the probe frequency by almost one cavity’s free spectral range (FSR ≈ 204.35 MHz) and beyond the cavity resonance to avoid its influence on the ring-down signals measurement and locking the laser to the cavity. Another AOM shifts the LO beam to set its detuning from the probe, $\nu_{L}-\nu_{P}$, constant through the measurement of the entire spectrum. Note that laser tuning in HCRDS systems can also be realized without B-EOM, by relocking the laser to subsequent cavity modes, at the cost of lower tuning speed. This approach would result in absorptive and differential dispersive spectrum (*20*).

To scan the molecular spectrum, the B-EOM modulation frequency is stepped in increments equal to the FSR. Our maximum scanning range when using the first-order sideband is ±20 GHz and can be further multiplied as the sideband order increases. For each frequency step, the single heterodyne ring-down signal is acquired. Frequency scanning through the molecular spectrum is repeated. For each frequency of the spectrum, corresponding to the center of the cavity mode, the power spectra of individual heterodyne light decays are averaged. This approach is preferred instead of averaging the decays due to their slow temporal phase changes. From these power spectra, the positions and widths of the cavity modes are retrieved. Further technical details are provided in the “Experimental design” section.

The cavity mode widths provide the HCRDS absorptive spectrum with absorption coefficient $\alpha \left(\nu_{C}\right)=4\pi ∆\gamma_{C}\left(\nu_{C}\right)\;c^{-1}$, where $∆\gamma_{C}=\gamma_{C}-\gamma_{C,0}={\left(4\pi \right)}^{-1}\mathrm{cA}\;\text{Re}\left\{ℒ\left(\nu_{C}\right)\right\}$, c is the speed of light, $\gamma_{C,0}$ is the cavity mode half-width in the absence of molecular absorption, A is an area under the spectral line, and ℒ(ν) is the normalized complex-valued line-shape function so that ∫ℒ(ν)dν=1. The HCRDS dispersive spectrum is obtained from the frequency shift $∆\nu_{D}\left(\nu_{C}\right)$ between cavity mode positions disturbed and undisturbed by the presence of the molecular resonances. The mode positions, shifted by a broadband nonresonant dispersion of the sample and the cavity mirrors, are fitted as a background of the dispersive spectrum. The dispersive spectrum is given by $∆\nu_{D}\left(\nu_{C}\right)={\left(4\pi n_{0}\right)}^{-1}\mathrm{cA}\;\text{Im}\left\{ℒ\left(\nu_{C}\right)\right\}$, where $n_{0}$ is a broadband refractive index of the sample. Note that the dispersive cavity mode shift $∆\nu_{D}$ and absorptive cavity mode width $∆\gamma_{C}$ (*19*) are related by the Kramers-Krönig (*32*) relation, which yields $∆\nu_{D}∆{\gamma_{C}}^{-1}=n_{0}^{-1}\text{Im}\left\{ℒ\left(\nu_{C}\right)\right\}/\text{Re}\left\{ℒ\left(\nu_{C}\right)\right\}$. Accurate measurement of the line area A at a known absorber concentration $N_{a}$ allows one to determine the line intensity $S=A/N_{a}$.

### HCRDS accuracy tests at the sub–per-mil level on CO line example

**Fig. 2.**
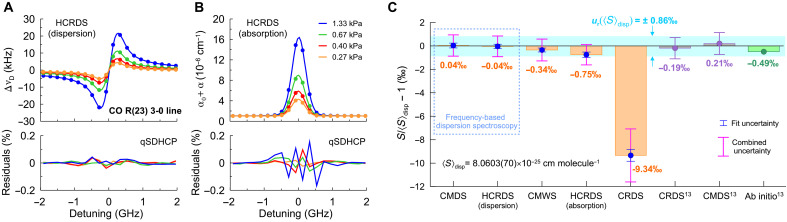
The accuracy of HCRDS. (**A** and **B**) Examples of dispersive (A) and absorptive (B) HCRDS spectra. Residuals below correspond to the difference between experimental and fitted qSDHCP line-shape model. The R(23) 3-0 line of ^12^C^16^O (${\tilde{\nu }}_{0}=6410.879558\;{\text{cm}}^{-1}$) was measured at temperature 296 K at pressures ranging from 0.27 to 1.33 kPa. Each of the spectra presented is an average of 5000 spectral scans, and the signal-to-noise ratio ranges from 1800:1 to 5000:1. Residuals are presented relative to the line profile amplitude at 1.33 kPa. Typical measurement time for one scan is 60 ms. The offset frequency of 192193.256628 GHz is subtracted from the abscissa. (**C**) Comparison of HCRDS results for the intensity of CO R(23) 3-0 line with CMDS, CMWS, and CRDS measurement results in this work as well as literature data (*13*) and ab initio calculation (*13*). The reference line intensity ${\langle S\rangle }_{\text{disp}}$ is the average of the dispersion measurement results with CMDS and HCRDS. These dispersive measurements provide the relative combined uncertainty of 0.86‰. Combined uncertainty includes both type A (fit uncertainty) and type B uncertainties. The large deviation seen for CRDS result is caused by the nonlinearity of the detection bandwidth. The remaining experimental results are in sub–per-mil agreement with each other as well as with the best reported data and the ab initio results.

Examples of HCRDS absorption and dispersion spectra of ^12^C^16^O transition are shown in Fig. 2 (A and B). As a benchmark transition, we chose one of the most accurately known molecular lines, R(23) from the 3-0 band, the line intensity of which was measured using several techniques with sub–per-mil accuracy (*13*). Moreover, because CO is a simple diatomic molecule, its line intensity can be calculated from first principles with high accuracy (*13*). Fitting the spectra with the quadratic speed-dependent hard-collision profile (qSDHCP) (*33*–*35*) results in the best agreement with the experimental line shape. The lowest SD of the fit residuals achieved for dispersion was 0.14 per mil (‰) and for absorption was 0.58‰. It should be noted that the qSDHCP is a variant of the now recommended line-shape model for atmospheric data analysis (*36*), and the demonstrated accuracy of the laboratory dispersion spectrum is an order of magnitude greater than that required for atmospheric studies. In Fig. 2C, CO line intensities recorded using HCRDS are compared with results provided by other techniques implemented here in parallel with HCRDS, as well as with literature data and the ab initio calculation. A comparison of HCRDS and CMDS dispersion techniques shows an excellent 0.04‰ agreement with their averaged value. The CO R(23) line intensity, 8.0603(70) × 10^−25^ cm molecule^−1^, reported here has a sub–per-mil relative combined uncertainty. Moreover, a comparison with CMDS data (*13*) from 3 years ago demonstrates the long-term repeatability of our dispersion measurements at 2 × 10^−4^ level. Absorption measurements using HCRDS and cavity mode-width spectroscopy (CMWS) (*37*) provide combined uncertainties similar to dispersive methods. They introduce a small bias in line intensity but within the range of combined uncertainty. This bias is expected because absorption measurements are susceptible to nonlinearity in light-intensity detection. This susceptibility is two orders of magnitude lower for the dispersion techniques (see the “Simulations” section). Measurement of CO transition by CRDS introduced a large bias, almost 1%, in the line intensity. Furthermore, replacing the optical detector in the detection system produced a different result. Research on the dependence of the line intensity on various configurations of the CRDS detection system has recently been carried out (*6*). The main reason for the discrepant results obtained with CRDS is the nonlinearity of the detection bandwidth. We found that choosing a linear detector response range in HCRDS can reduce this nonlinearity-related error by over seven orders of magnitude (see the “Simulations” section). Except for CRDS, all of our CO line intensity results agree with each other in the sub–per-mil range. Moreover, our dispersion results agree up to 0.2‰ with the best CO experimental data published to date (*13*) and up to −0.49‰ with the best ab initio calculation of the CO line intensity (*13*).

### Per-mil–level accuracy spectroscopy of HD transition

**Fig. 3.**
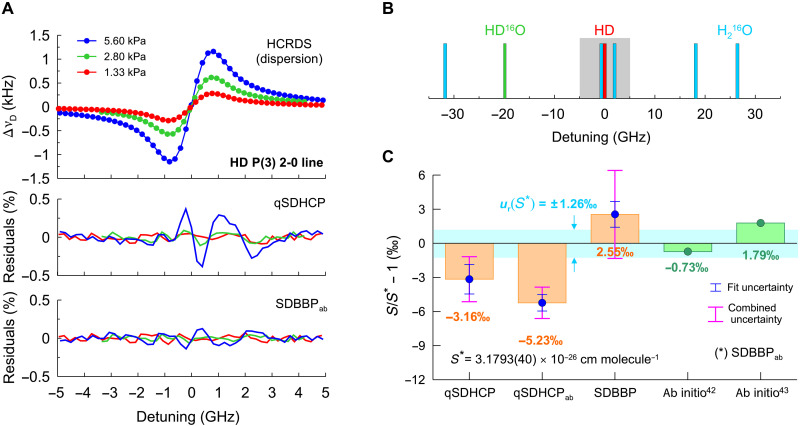
Per-mil–level accuracy spectroscopy of the molecular hydrogen line shape. (**A**) The dispersive HCRDS spectrum of the P(3) 2-0 line of HD (${\tilde{\nu }}_{0}=6798.7679\;{\text{cm}}^{-1}$) measured at temperature 296 K at pressures ranging from 1.33 to 5.60 kPa. The residuals below correspond to the difference between the experimental model and the fitted line-shape model. In fits, qSDHCP_ab_ and SDBBP_ab_ were used. The subscript ab indicates that line-shape parameters such as collisional width and shift, Dicke narrowing, and speed dependence of collisional width and shift were set to the ab initio values. Each spectrum is an average of 5000 to 15,000 spectral scans, and the signal-to-noise ratio ranges from 700:1 to 2000:1. Residuals are presented relative to the line profile amplitude at 5.60 kPa. Typical measurement time for one scan is 750 ms. The offset frequency of 203821.934011250 GHz is subtracted from the abscissa. (**B**) Transitions in H_2_^16^O and HD^16^O molecules that were included in the analysis of the HD P(3) 2-0 line. The rectangle indicates the spectral range of the HD line measurement. (**C**) Line intensity measurement results of the HD spectrum from (A) and their comparison with the ab initio results (*42*, *43*). The reference line intensity S* corresponds to SDBBP_ab_ analysis with a relative combined uncertainty of 1.26‰. Combined uncertainty includes both type A (fit uncertainty) and type B uncertainties. Results of analysis using qSDHCP, qSDHCP_ab_, and SDBBP are also shown. Per-mil agreement with the ab initio intensity and shape of the HD line is presented.

Dispersive HCRDS spectroscopy reveals its potential for the fundamental studies of collisions between hydrogen molecules through accurate measurements of the shape of its spectral lines. In Fig. 3A, we show the HCRDS dispersion spectrum of the P(3) 2-0 band HD transition. The qSDHCP does not fully describe the experimental spectrum for this molecular system, which is revealed as a systematic structure on the fit residuals. For lighter molecules such as HD, attention needs to be paid to the proper description of the velocity-changing collisions leading to Dicke narrowing of the line. The speed-dependent billiard-ball profile (SDBBP) (*38*) provides a more physical description of molecular collisions and allows the HD spectrum to be modeled down to random noise in residuals with a SD of 0.6‰. Calculations of SDBBP at low pressures used here require the implementation of an iterative approach (*39*). We note that the selected HD transition, unlike the CO transition, is weaker and not so well isolated from other transitions (Fig. 3B). Our results for the HD line intensity and their comparison with the theory are shown in Fig. 3C. The reference is the line intensity of 3.1793(40) × 10^−26^ cm molecule^−1^ obtained from the SDBBP analysis, with a relative combined uncertainty of 1.26‰, in which the line-shape parameters were fixed to ab initio values calculated in this work (see the “Ab initio calculations” section). If the line-shape parameters were fitted, then SDBBP gave a small deviation of 2.55‰ from the reference line intensity. The results for the qSDHCP show a systematic bias of up to −5.23‰ compared to the reference value due to the incorrect model used to analyze the experimental spectrum. The HD line intensity reported here is one of the most accurate experimental results to date (*40*, *41*). Moreover, its comparison with ab initio calculations of line intensity provided by (*42*) and (*43*) gives excellent agreement up to −0.73‰ and 1.79‰, respectively. Analysis of the unperturbed line position revealed that the qSDHCP leads to 7.63 MHz higher result compared to the SDBBP fit. We not that the latter one is only 0.49 MHz (*36*) higher than ab initio QED calculations for the HD line position from (*44*). This is 0.25‰ agreement compared to the 2-GHz-wide Doppler-broadened molecular resonance.

## DISCUSSION

In this work, we raise two critical issues. First, we show that the popular, ultrasensitive, and high-precision CRDS technique can also achieve substantially improved accuracy if only the optical frequency information carried by the ring-down electromagnetic field is exploited. We propose a heterodyne detection scheme that, if applied to the CRDS configuration, will enable the acquisition of dispersive spectra with sub–per-mil accuracy, robust to distortions caused by the nonlinearity of light intensity measurements. Second, we demonstrate that our pure frequency–based dispersive spectroscopy technique has a noticeable advantage over all traditional intensity–based techniques in terms of high fidelity of the acquired molecular spectra. Our research represents an essential step toward improving those research areas where accurate measurement of the shape and intensity of molecular lines is crucial and where intensity-based techniques, including CRDS, are widely used. The most representative example is atmospheric research, including spatial and temporal monitoring of GHGs, forecasting future climate changes, and examining the dynamics and kinetics of processes in the atmospheric layers. In all these studies, to minimize systematic errors in the analysis of spectroscopic retrievals, the accuracy requirements imposed on the spectroscopic parameters of the database are at the level of 1‰. As a reminder, most CRDS systems can provide reference data with a typical accuracy of no better than 10‰. Our technique exceeds 1‰ accuracy.

A very insidious systematic error of the traditional CRDS may come from the distortion of the detection band that modifies the decay time constant but not the exponential shape of the decay. In such cases, we see flat residuals from the exponential fit to the decay but we are completely unaware of the systematic error that we are making. A recently proposed solution for the conventional CRDS recommends calibration of its detection system (*6*). Although this ultimately provides accuracy similar to the dispersive HCRDS presented here, the calibration process can only be performed in a few metrology institutes worldwide. Our dispersive HCRDS technique does not require calibration, and achieving high accuracy in measurements is much more convenient. In principle, it does not require complicated modifications to existing CRDS systems with frequency stabilization. With the appropriate selection of the linear range of the detection band, this technique is practically insensitive to detection band nonlinearities. It should be noted that the CBDS technique may have a similar insensitivity to detector band nonlinearity, but the selected detector band frequency results from the choice of the excitation laser detuning from the cavity mode. Therefore, in CBDS, selecting higher frequencies in the detector band will always result in greater attenuation of the signal transmitted through the cavity. Dispersive HCRDS is also two orders of magnitude less susceptible to signal amplitude nonlinearity than traditional CRDS. Moreover, because the HCRDS dispersion spectrum is entirely measured in frequencies, it can be related to an atomic frequency standard. Such an approach ensures traceability to the International System of Units (SI) and can notably facilitate the comparison of interlaboratory data. All these features are unattainable with traditional CRDS.

Analyzing the uncertainty budget of determined in this work line intensities (see the “Statistical analysis” section), it can be seen that the main contribution comes from pressure measurement and the uncertainty of the sample composition. This provides the unique opportunity to use the dispersive HCRDS technique to develop new optical standards for gas composition and pressure based on the measurement of the spectral line intensity. Gas pressure measurement based on the resonant refractive index (i.e., spectroscopic measurement), rather than nonresonant as in current cavity-based refractometry, has the advantage of being much less sensitive to cavity deformation and diffraction effects (Gouy phase shift) (*9*). These factors affect the shift of the entire comb of cavity modes, in addition to the pressure-induced changes. Although refractometric measurements of cavity mode positions relative to their vacuum positions pose a challenge, this issue does not affect the measurement of local shifts in cavity modes near molecular resonances. We expect that further development of ab initio line intensity calculations will drive progress in this field.

A substantial contribution to the uncertainty budget of the line intensity also comes from choosing the correct line-shape model. Accurate dispersive HCRDS spectra can help in testing theoretical models of intermolecular interactions and ab initio calculations of line intensities and line-shape parameters. It appeared that variant of the now recommended standard line-shape model for the new generation of spectroscopic databases (*36*, *45*), the qSDHCP profile, is no longer sufficient to describe the shape of molecular hydrogen lines accurately. We have shown that more physical SDBBP should be used instead. The intensity of the HD line obtained using this model, combining ab initio line-shape parameters, agrees excellently with ab initio–calculated line intensity. As a result, dispersive HCRDS confirmed both the ab initio intensity and the ab initio shape of the molecular hydrogen line, reaching per-mil accuracy. Moreover, the use of the SDBBP eliminates a few MHz error introduced by the qSDHCP in determination of the HD line position what is crucial in the fundamental study of QED in the molecular systems.

The results from Fig. 2C clearly show that agreement between the current most accurate experimental results for the CO line intensity is better than their agreement with the most accurate ab initio result. Therefore, line intensities determined by our method can serve as a sub–per-mil reference used in such applications as global GHG measurements, studies of the atmosphere of exoplanets, spectroscopic metrology of isotope ratios, or monitoring trace humidity in semiconductor production. Last, the presented dispersive spectroscopy stimulates research devoted to accurate ab initio calculations of spectral line shapes and their intensities, which have implications for fundamental science and many applications.

Like any other technique, also ours has some limitations, and some aspects need to be addressed in future applications. First, the current state of the HCRDS system allows to achieve a measurement sensitivity of 8.7 × 10^−12^ cm^−1^ (see the “Sensitivity” section) compared to the best sensitivities of 1.0 × 10^−14^ − 3.5 × 10^−12^ cm^−1^ offered by cavity-enhanced methods, which additionally use such technologies as high-quality isolation of the cavity from mechanical and acoustic vibrations of the environment (*46*), high-frequency modulation techniques for sensitive detection (*47*), or differential absorption measurements minimizing effects of etalons (*48*). This, however, does not change the fact that the sensitivity of HCRDS, especially its dispersive part, is susceptible to frequency fluctuations of the LO beam, which are caused by the fluctuating offset of the error signal in the Pound-Drever-Hall (PDH) locking scheme of the laser to the cavity (see the “Sensitivity” section). Further progress in the development of the HCRDS sensitivity will require an active compensation for the residual amplitude modulation effect in the locking servo loop in a similar manner to (*49*). Second, the now determined dynamic range of the HCRDS method (see the “Sensitivity” section), provided that its per-mil measurement precision is not lost, is 1.8 × 10^6^ and 2.4 × 10^5^ for absorptive and dispersive HCRDS, respectively. It is interesting to see whether it is possible to measure higher absorptions than 1.6 × 10^−5^ cm^−1^ without loss of precision. We have already shown that the dynamic range of CMDS can be 10 times larger than CRDS and reach an absorption of 1.0 × 10^−4^ cm^−1^ (*21*). Insensitivity of HCRDS to the nonlinearity of the detection band should provide a noticeable advantage over CRDS. It should be noted that this nonlinearity becomes particularly problematic as absorption increases, both due to the shortening of the light decay and the narrowing of the detector bandwidth resulting from the need for higher gain, thus limiting the dynamic range of any conventional intensity-based CRDS. Shorter decays mean wider cavity mode widths, which is not a problem, but rather an advantage for HCRDS, but the limiting factor may be the finite bandwidth of the dc detector (we assumed here that variations of the absorption coefficient are insignificant within the range of the cavity mode width). The solution for HCRDS may be the use of AC detectors and consistently adjust the LO-probe beating frequency to the flat-response detector range whenever increasing absorption requires increasing detector gain. Third, the small systematic structure visible in the residuals for the CO line, see Fig. 2A (qSDHCP fit), requires further investigation. Using a more physical billiard ball profile may improve results for the CO transition, if only ab initio calculations of CO line-shape parameters would be available. For the HD line, one should check whether selecting an adjacent HD line that is less affected by water lines will produce equally accurate results.

In conclusion, we have developed the heterodyne, frequency-based dispersive cavity ring-down technique that exploits optical cavity eigenmodes to provide high-fidelity molecular spectra with sub–per-mil accuracy. This method is a key step toward improving the accuracy of many spectroscopy-based research fields, such as fundamental studies of QED in molecular systems, atmospheric remote sensing, isotope ratio metrology, and the establishment of spectroscopic pressure, temperature, and amount of substance gas standards based on quantum properties of molecules.

## MATERIALS AND METHODS

### Experimental design

**Fig. 4.**
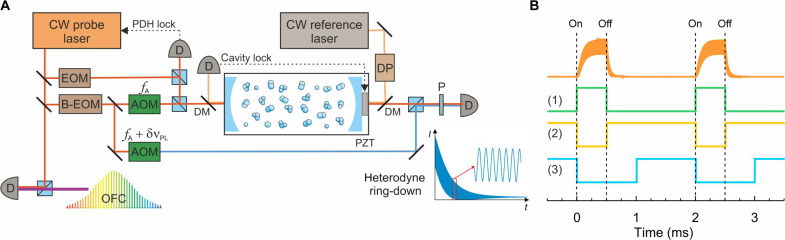
HCRDS experiment. (**A**) A continuous-wave (CW) probe laser is locked to the cavity by the Pound-Drever-Hall (PDH) method. An electro-optic modulator (EOM) modulates the laser light phase to generate the PDH error signal. The cavity length is stabilized with respect to another CW reference laser. The absolute frequency of the laser is measured using the optical frequency comb (OFC). The intracavity gas sample is probed using a single sideband of the B-EOM scanning in the microwave range. Acousto-optical modulators (AOM) are used to detune the carrier frequency from the cavity resonance by $f_{A}$ and also to detune the LO beam from the probe beam by $\delta \nu_{\text{PL}}$. The inset presents an example of a heterodyne ring-down signal. Explanation of abbreviations: D, photodetector; DM, dichroic mirror; PZT, piezo transducer; OFC, optical frequency comb; P, polarizer; DP, double-pass AOM system. (**B**) Top: Heterodyne response of the cavity to periodic laser power switching. Bottom: Time sequences of TTL signals used in the HCRDS experiment to trigger ring-down signals (1), to start the data acquisition process (2), and to determine the frequency switching time of the microwave generator controlling B-EOM (3).

A sketch of the experimental system implementing the HCRDS method is shown in Fig. 4A. To measure the shapes of the CO and HD lines, two external cavity diode lasers (Toptica CTL) were used, covering the spectral ranges of 1520 to 1630 nm and 1460 to 1570 nm, respectively. In each case, the laser beam was divided into two orthogonally polarized beams. An s-polarized beam, phase-modulated at 20 MHz by a narrowband electro-optic modulator (EOM), was used to lock the laser to the optical cavity using the PDH scheme (*50*). The frequency stability of this lock was at the hertz level. A small portion of the p-polarized laser beam was transmitted via optical fiber to an optical frequency comb system (Menlo Systems) to enable continuous measurement of the absolute laser frequency. The main part of the p-polarized beam was further split into two beams: one for excitation of the optical cavity mode and the other for the reference heterodyne detection of light decays from the cavity. Typical power of the LO beam was ~8 μW and was the same for the probe beam leaving the cavity when there was no absorbing medium in it. Both beams were frequency stepped using a B-EOM and a microwave driver (*25*). Although such configuration generates a series of sidebands on the laser, the optical cavity acts as a spectral filter, allowing only one sideband of the excitation beam to resonate with the cavity. Similarly, in the case of heterodyne detection, the limited detection system bandwidth allows the observation of the decay of the beat signal with only one sideband of the reference beam. We chose a first-order sideband that enables scanning of the excitation and reference beams in the frequency range of 0.2 to 20 GHz. Note, however, that the scanning range can be further multiplied by choosing a higher-order sideband. The ring-down decays were initiated after turning off the excitation beam by an AOM driven by frequency $f_{A}$. This AOM also shifts the carrier frequency by 200 MHz, which is sufficiently far from the cavity FSR of 204.35 MHz, to avoid its influence on the measurement of ring-down signals and locking the laser to the cavity. Another AOM detunes the frequency of the reference beam from the frequency of the excitation beam by a constant value of $\delta \nu_{\text{PL}}$ equal to several megahertz.

The optical cavity was formed by two high-reflectivity mirrors having 1-m radii of curvature and placed at a distance of ~73 cm from each other. The mirrors are dual-wavelength coated to use a separate laser (wavelength of 1064 nm and mirrors reflectance of about 0.98) to stabilize the cavity length (*51*). This was done with respect to the I_2_-stabilized Nd:YAG (Innolight Prometheus P20) laser characterized by short-term frequency stability of ~2 kHz and relative long-term frequency stability of 4 × 10^−14^ after 100 s of averaging (*52*). The double-pass AOM system (*53*) and phase-sensitive detection were used to generate an error signal in the cavity length locking loop servomechanism. Two sets of cavity mirrors were used for HCRDS measurements: one for the CO molecular system and the other for HD. They were characterized by a reflectance coefficient of 0.999925 and 0.999993, corresponding to full widths at half maximum of the cavity mode of 4.9 and 0.43 kHz, respectively. They lead to cavity finesse of 41,900 and 449,000 and empty cavity ring-down time constants of 32 and 350 μs, respectively. Because the high-reflectance optical coatings of cavity mirrors are usually nonuniformly stressed during the deposition process, this can lead to residual birefringence of the mirrors. It should be noted that, as in the case of CRDS, the residual mirror birefringence may cause the bi-exponentiality of the light decay (*54*–*56*), in HCRDS, it is responsible for the asymmetry of the cavity mode shape (bi-Lorentzian), which affects the accuracy of determining both the position and width of the cavity mode. To reduce this effect, we placed a super-achromatic wave plate in front of the cavity entrance and precisely aligned the light’s polarization axis with the mirror’s birefringence axis. After this treatment, we no longer observed any distortions in the shape of the Lorentzian cavity mode caused by the mirror birefringence effect.

In R(23) 3-0 CO transition measurements, we used a commercial sample of CO (Linde Gas) with purity of 0.99997, produced by the reaction of water with petrogenic natural gas having an estimated ^13^δC_VPDB_ isotope content of −40‰ with respect to the Vienna Pee Dee belemnite scale (*3*). In P(3) 2-0 HD transition measurements, we used a commercial HD sample (Sigma-Aldrich) containing 97.0% of HD, 1.7% of H_2_, and 1.2% of D_2_. During the measurements, the sample gas pressure was measured in parallel using three manometers (MKS Baratron 690A with 1.33- and 13.3-kPa full range and Mensor CPG2500 with 120-kPa full range) with the highest relative combined uncertainty of the absolute pressure measurement of 6 × 10^−4^. The cavity temperature was stabilized at 296 K with a total standard uncertainty of 30 mK. Analog ring-down decays, measured with a dc-coupled photodetector (New Focus 2053) with a bandwidth of up to 7 MHz, were digitized with an oscilloscope card characterized by a bandwidth of 100 MHz and 14-bit vertical resolution (National Instruments PCI-5122).

To scan the molecular transition, the B-EOM modulation frequency was stepped in increments of the FSR. A list of necessary frequencies was prepared and loaded into the microwave generator’s memory before each spectrum measurement for fast frequency switching. Then, the TTL signal controlled the determination of subsequent frequencies at the generator output. The time sequences of the TTL signals used in the experiment to trigger the ring-down, acquire its signal, and switch the microwave frequency are shown in Fig. 4B. The typical TTL signal period was ~2 ms for CO measurements and ~15 ms for HD measurements. During this time, the heterodyne decay signal was acquired, the frequency of the microwave generator was changed, and the cavity was pumped with light at the next frequency. The frequency switching time of the microwave generator was <1 ms. The size of the frequency list loaded into the microwave generator’s memory allowed the entire spectrum to be measured several dozen times without interruption. After collecting the appropriate amount of data, the initial averaging process began. For each frequency in the spectrum, the power spectra, not the decays themselves, were averaged because of slow phase changes in the collected heterodyne light decays for that frequency. From the averaged power spectra of heterodyne ring-down signals, positions and widths of cavity modes were fitted, and, from them, the absorption and dispersion spectra were obtained. For CO measurements, 5000 spectral scans were collected for each pressure, while, for HD, the number of scans was between 5000 and 15,000. The absolute frequency axis of molecular spectra and high stability of frequency references was provided by the mode-locked optical frequency comb and link to the UTC(AOS) (Coordinated Universal Time from the Astro-Geodynamic Observatory in Borowiec, Poland) frequency standard, respectively (*57*).

### Simulations

To gain a comprehensive understanding of how distortions in the ring-down decay signals, induced by the nonlinearity of light intensity detection, affect the accuracy of determined spectral line intensities, it was necessary to initially model the temporal response of the cavity to laser beam deactivation. Subsequently, we simulated conditions close to the experimental ones, in which nonlinear light detection effects modify the measured intensity of the light, which emerges from the cavity. In the following calculations, we neglect the laser jitter and ignore the finite bandwidth of the detection system.

Let us consider the excitation of the optical cavity with a light electric field $E_{i}\left(t\right)$, which is turned on abruptly at the time t=0, i.e., $E_{i}\left(t\right)=E_{i}^{0}e^{-i\omega t},t\geq 0$ and $E_{i}\left(t\right)=0,t<0$. By abrupt turning on the light, we mean that the condition $\Gamma_{C}\ll \Gamma_{0}\ll 2\pi /t_{r}$ is satisfied, where $\Gamma_{C}=-t_{r}^{-1}\;\text{ln}\left(Re^{-\alpha L}\right)$ is the angular frequency related to the measured cavity mode half width at half maximum $\gamma_{C}={\left(2\pi \right)}^{-1}\Gamma_{C}$, $\Gamma_{0}$ is the turn-on rate of the laser field, and $t_{r}=2L/c=\delta_{\text{FSR}}^{-1}$ is the round-trip time in the cavity where $\delta_{\text{FSR}}$ is the cavity FSR. Under this assumption, the field leaving the cavity at a given time t is a finite sum of components oscillating at the input frequency ω, corresponding to *M* round trips in the cavity completed to this moment (*58*)

$$E_{\text{out}}^{\text{ON}}\left(t\right)=\left(1-R\right)e^{-\alpha L/2}\sum_{m=0}^{M}R_{\text{eff}}^{m}E_{i}\left(t-mt_{r}\right)$$ (2)

where R is the geometric mean reflectance of cavity mirrors, L is the cavity length, α is the absorption coefficient of the intracavity gas sample, and $R_{\text{eff}}=Re^{-\alpha L}$ is an effective cavity mirrors reflectivity. Further expansion of Eq. 2 leads to the finite geometric series

$$E_{\text{out}}^{\text{ON}}\left(t\right)=\left(1-R\right)e^{-\frac{\alpha L}{2}}E_{i}\left(t\right)\sum_{m=0}^{M}{\left(R_{\text{eff}}e^{i\delta \omega t_{r}}\right)}^{m}$$ (3)

In the transition from Eq. 2 to Eq. 3, we represented the electric field angular frequency as $\omega =\omega_{C}+\delta \omega$, where δω describes detuning of ω from the cavity mode angular frequency $\omega_{C}=2\pi qt_{r}^{-1}$ and q is the cavity mode number. This allowed us to replace the expression $e^{-i\omega t_{r}}$ by $e^{-i\delta \omega t_{r}}$ because $e^{-i\omega_{C}t_{r}}=1$. The form of Eq. 3 shows the phase shifts experienced by the different components of the finite series as they pass through the cavity, which result from the mismatch of the laser and cavity frequencies. By calculating the geometric series in Eq. 3 and replacing the discrete time values $mt_{r}$ with a continuous quantity t (due to small $t_{r}$ values), one can represents with a good approximation that the field leaving the cavity as a superposition of the instantaneous laser field at the input frequency ω and the cavity response field centered around the cavity mode frequency ω_C_ and decaying with a rate of Γ_C_ (*21*)

$$E_{\text{out}}^{\text{ON}}\left(t\right)=E_{\text{out}}^{0}\frac{\Gamma_{C}^{0}}{\Gamma_{C}-i\delta \omega }\left(e^{-i\omega t}-e^{-\Gamma_{C}t}e^{-i\omega_{C}t}\right)$$ (4)

In the above, $E_{\text{out}}^{0}=E_{i}^{0}e^{-\alpha L/2}$, and $\Gamma_{C}^{0}\approx t_{r}^{-1}\left(1-R\right)$ is Γ_C_ for α = 0. It should be noted that the full agreement between Eqs. 3 and 4 occurs only for discrete times ${\mathrm{mt}}_{r}$. In turn, the form of Eq. 4 based on interfering fields provides a convenient and intuitive description of the interference observed experimentally on the cavity transient response under conditions of no frequency matching of the laser and cavity fields (δω ≠ 0) and before the steady state is reached in the cavity for *t* → ∞ (*29*, *31*). This approximation is valid, however, for signal detection time window $\delta t\gg t_{r}$, which, in practice, we often deal with. The complex Lorentz function ${\left(\Gamma_{C}-i\delta \omega \right)}^{-1}$ in Eq. 4, characterized by α-dependent width Γ_C_, relates to the cavity mode shape and quantifies the fraction of the laser field, which is transmitted through the cavity when the laser frequency is detuned by δω away from the cavity mode center.

Now, let the laser field be turned off at time $t_{0}$ corresponding to M round trips in the cavity made to that moment, i.e., $t_{0}=Mt_{r}$. We assume a smooth switch off the laser field with a finite switch-off time constant $\Gamma_{0}^{-1}$ (*30*) and with an infinitely large extinction ratio. In the time domain, the laser field is

$$\begin{array}{cc} E_{i}\left(t\right)=0 & t<0\\ E_{i}\left(t\right)=E_{i}^{\prime }\left(t\right)=E_{i}^{0}e^{-i\omega t} & 0\leq t\leq t_{0}\\ E_{i}\left(t\right)=E_{i}^{\prime \prime }\left(t\right)=E_{i}^{0}e^{-\Gamma_{0}\left(t-t_{0}\right)}e^{-i\omega t} & t>t_{0} \end{array}$$ (5)

The exponential model of turning off the laser beam allows for simplified calculations. The field leaving the cavity after N additional cavity round trips counted from the moment the laser beam is turned off, i.e., after time $t=t_{0}+Nt_{r}$, can be presented as a two finite sums describing processes of building the field in the cavity (before time $t_{0}$) and the decay of the field from the cavity (after time $t_{0}$)

$$E_{\text{out}}^{\text{OFF}}\left(t\right)=\left(1-R\right)e^{-\alpha L/2}\left[\sum_{m=N}^{N+M}R_{\text{eff}}^{m}E_{i}^{\prime }\left(t-mt_{r}\right)+\sum_{m=0}^{N-1}R_{\text{eff}}^{m}E_{i}^{\prime \prime }\left(t-mt_{r}\right)\right]$$ (6)

As before, we use $\omega =\omega_{C}+\delta \omega$ and replace all expressions $e^{-i\omega {\mathrm{jt}}_{r}}$ with $e^{-i\delta \omega t_{r}}$, where j is an integer. With the same assumptions as before, we move from discrete time values $jt_{r}$ to continuous quantity t. After calculating the geometric series in Eq. 6, we obtain the field passing through the cavity in two parts

$$\begin{array}{c} E_{\text{out}}^{\text{OFF}}\left(t\right)=\\ E_{\text{out}}^{0}\;e^{-i\omega t_{0}}\Gamma_{C}^{0}\left\{\frac{1-\left(1-\Gamma_{C}/\Gamma_{0}+i\delta \omega /\Gamma_{0}\right)e^{-\left(\Gamma_{C}-i\delta \omega \right)t_{0}}}{\left(1-\Gamma_{C}/\Gamma_{0}+i\delta \omega /\Gamma_{0}\right)\left(\Gamma_{C}-i\delta \omega \right)}\times \right.\\ \left.e^{-\left(\Gamma_{C}+i\omega_{C}\right)\left(t-t_{0}\right)}-\frac{1}{\Gamma_{0}-\Gamma_{C}+i\delta \omega }e^{-\left(\Gamma_{0}+i\omega \right)\left(t-t_{0}\right)}\right\} \end{array}$$ (7)

where the first part describes the exponential decay of the cavity mode field with the time constant $\Gamma_{C}^{-1}$ and the second part is the adopted model of the exponential decay of the laser field. Equation 7 is consistent with equation 10b of (*30*) apart from the term $\left(1-\Gamma_{C}/\Gamma_{0}+i\delta \omega /\Gamma_{0}\right)e^{-\left(\Gamma_{C}-i\delta \omega \right)t_{0}}$, indicating that, in our calculations, the steady state of the cavity field is not achieved before time $t_{0}$, while, in (*30*), the description of the cavity decay begins after reaching the steady state. The amplitude of the laser field passing through the cavity depends on the detuning δω of the laser from the center of the cavity mode, the profile of which is defined as before by the complex Lorentz function, but with a width modified to $\Gamma_{0}-\Gamma_{C}$. If the laser light is turned off very quickly such that $\Gamma_{0}\left(t-t_{0}\right)\gg 1$, then the first term in Eq. 7 dominates and the expression for $E_{\text{out}}^{\text{OFF}}\left(t\right)$ can be reduced to a simple single-exponential decay form

$$E_{\text{out}}^{\text{OFF}}\left(t\right)=E_{\text{out}}^{\text{ON}}\left(t_{0}\right)e^{-\left(\Gamma_{C}+i\omega_{C}\right)\left(t-t_{0}\right)}$$ (8)

However, if the laser turn-off rate Γ_0_ is relatively small, then Eq. 7 clearly shows that the recorded light decay will be distorted as a result of interference at the frequency δω between the laser and cavity fields. Please note that, even if the laser field is turned off quickly enough, but the extinction ratio of the laser field amplitude is finite and relatively low, e.g., 50 dB, light oscillations will appear at the cavity output when δω ≠ 0, because the residual laser field will interfere with a decaying cavity field. This effect has already been observed experimentally (*29*), and its contribution to systematic distortions of ring-down signals has also been investigated (*59*). Heterodyne, frequency-domain detection of ring-down signals in HCRDS has an advantage over time-domain CRDS because it can easily remove from the Fourier transform spectrum the sharp peak at frequency $\nu_{L}-\nu_{P}$ corresponding to the interference between the residual probe and the LO fields. We note that, in our CRDS and HCRDS measurements, we were far from the limits discussed above because $\Gamma_{0}$ was more than 600 times larger than $\Gamma_{C}$ and the extinction ratio of the laser field was ~80 dB.

In the HCRDS method, the ring-down decay field described by Eq. 8 is beaten with the laser field of the LO. The field incident on the detector is

$$E_{\text{out}}^{\text{OFF}}\left(t\right)=E_{\text{out}}^{\text{ON}}\left(t_{0}\right)e^{-\left(\Gamma_{C}+i\omega_{C}\right)\left(t-t_{0}\right)}+E_{L}e^{i\varphi_{L}}e^{i\omega_{L}\left(t-t_{0}\right)}$$ (9)

where $E_{L}$, $\omega_{L}$, and $\varphi_{L}$ are the amplitude, angular frequency, and phase of the LO field, respectively. The corresponding light intensity $∼{|E_{\text{out}}^{\text{OFF}}|}^{2}$ is

$$I_{\text{out}}\left(t\right)=I_{L}+I_{C}e^{-2\Gamma_{C}t}+2\eta \sqrt{I_{L}I_{C}}e^{-\Gamma_{C}t}\text{cos}\left({\delta \omega }_{\text{CL}}t+\varphi_{L}\right)$$ (10)

where ${\delta \omega }_{\text{CL}}=\omega_{C}-\omega_{L}$ is the beat frequency between the cavity and LO fields and η is the mode matching factor between the LO and decaying light beams. If $\omega_{L}$ is known, then the cavity mode position can be easily calculated from the measured value of ${\delta \omega }_{\text{CL}}$. We note that the measurement of the absolute $\omega_{L}$ frequency is not needed when both $\omega_{C}$ and $\omega_{L}$ are measured relative to the cavity mode to which the probe laser is locked (see Fig. 1C). Analysis of the Lorentz peak at the frequency ${\delta \omega }_{\text{CL}}$ in the PS of the light intensity $I_{\text{out}}$ provides information about both the dispersive shift of the cavity mode and its broadening due to absorption.

**Fig. 5.**
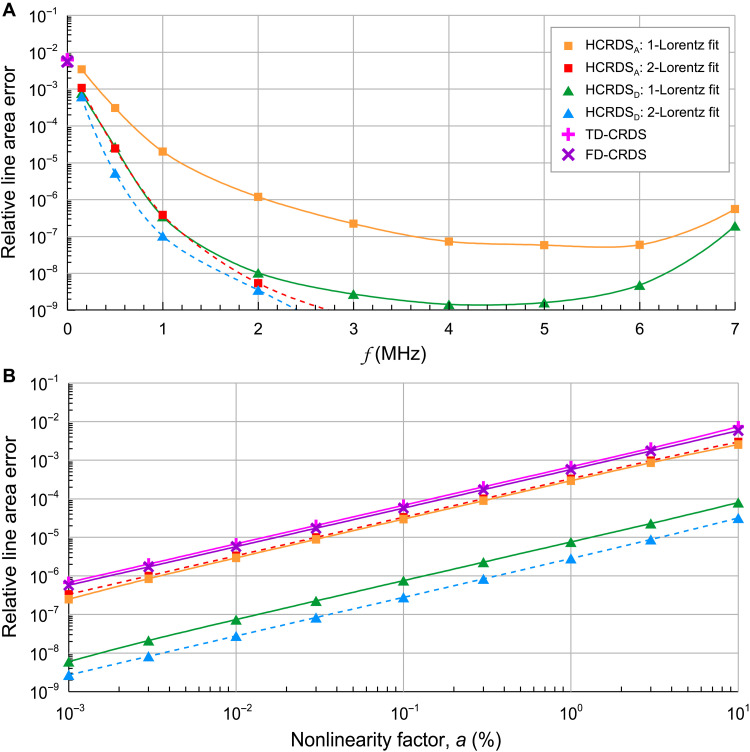
Nonlinearity of light detection. (**A**) The influence of the detection system frequency response, from Fig. 1B, on the accuracy of the line area determined from the simulated absorption and dispersion line profiles in HCRDS and CRDS. The 𝑓 axis corresponds to the beat frequencies between the LO laser and the cavity response (for CRDS, f=0). HCRDS results are presented for both absorption (HCRDS_A_) and dispersion (HCRDS_D_). Data marked as 1-Lorentz and 2-Lorentz correspond to different models used in the analysis of power spectra of heterodyne ring-downs. Results for traditional CRDS were obtained from both time (TD-CRDS) and frequency (FD-CRDS) domain analysis. (**B**) The influence of the nonlinearity of the detection system amplitude on the accuracy of the line area determined from simulated absorption and dispersion line profiles in HCRDS and CRDS. Results for different values of the nonlinearity parameter, *a*, are presented.

The TF of the detection system of the spectrometer (see the “Experimental design” section) was measured as its amplitude response to changes in the beat signal frequency of the detected light. We developed the TF model as a linear combination of complex-valued high-pass and low-pass filter functions. The parameters of this model were selected to reproduce best the experimental shape of the TF shown in Fig. 1B. To test the line intensity accuracy against the nonlinearity of the detection system’s bandwidth, the absorption and dispersion profile was simulated with the line intensity and shape parameters corresponding to the R(23) 3-0 CO transition at a pressure of 1.33 kPa. The light decay signal was simulated using Eq. 10 for each frequency and Fourier transformed. The result was multiplied by our complex-valued TF model and inverse Fourier transformed to reproduce the ring-down decay having a different shape than the original exponential one. The power spectra of the reproduced heterodyne ring-down decays were then analyzed, focusing on the frequency range around the frequency ${\delta \omega }_{\text{CL}}$. Two models, a single Lorentz profile centered on the frequency ${\delta \omega }_{\text{CL}}$ and two Lorentz profiles centered on ${\delta \omega }_{\text{CL}}$ and zero frequencies, were included in the analysis to provide the beat frequency parameter ${\delta \omega }_{\text{CL}}$ (for HCRDS dispersion) and cavity mode width (for HCRDS absorption). Last, the line area determined from the obtained absorption and dispersion profiles was compared with the simulated value, and the systematic deviation from it was calculated. The entire procedure was repeated for several beat frequencies up to 7 MHz. The results are shown in Fig. 5A. In the same figure, we also present results for CRDS. The procedure for simulating distorted light decays was similar to that described above for HCRDS, starting with single-exponential decay signals. Their analysis was performed both traditionally in the time domain, determining the time constant, and in the frequency domain using the Lorentz peak of the PS on the zero frequency to determine the cavity mode width. The results for HCRDS are clearly better than those for CRDS for all frequencies in the tested range of ${\delta \omega }_{\text{CL}}$. Note that the greatest advantage of HCRDS over CRDS at the relative accuracy level 10^−7^ is visible for the most flat region of the detection system frequency response (shown in Fig. 1B) around the frequency of 4 MHz. Furthermore, dispersive HCRDS, with an accuracy level > 10^−9^, appears to be more accurate than absorptive one. Using the model with simultaneous fitting of two Lorentz profiles further improves the accuracy of the retrieved line intensity, especially in the case of absorptive HCRDS.

In tests of the line intensity accuracy in terms of the nonlinearity of the detection system amplitude, the heterodyne ring-down decays simulated using Eq. 10 were multiplied by the nonlinearity function (*31*) $y\left(t\right)=1-aI_{\text{out}}\left(t\right)/I_{\text{out}}^{\text{max}}$, where $I_{\text{out}}^{\text{max}}$ is the maximum amplitude of $I_{\text{out}}\left(t\right)$ and a is the amplitude nonlinearity parameter in %. As before, the heterodyne ring-down decays were simulated at each frequency in the simulated CO spectrum. Absorption and dispersion profiles were obtained from them, and the determined line area was compared with the simulated one. The results are shown in Fig. 5B. In the simulation for HCRDS, we chose ${\delta \omega }_{\text{CL}}=4\;\text{MHz}$. To analyze the Lorentz peak in the PS, as before, both the single Lorentz profile centered on the frequency ${\delta \omega }_{\text{CL}}$ and two Lorentz profiles centered on ${\delta \omega }_{\text{CL}}$ and zero frequencies were used. The CRDS results included the decay signal analysis in both time and frequency domains. From Fig. 5B, it can be seen that the method of analyzing the light decays in CRDS does not affect the dependence of the line intensity accuracy on parameter a. HCRDS absorption results are similar to CRDS results. However, for dispersive HCRDS, we observe a two-order improvement in line intensity accuracy compared to CRDS and absorption HCRDS. This immunity to nonlinear detection is due to the fact, that any deformations of the Lorentz peak amplitude, but symmetrical about its center, will not affect its position. As before, the 2-Lorentz fitting model further improves the line intensity accuracy.

### Ab initio calculations

Quantum scattering calculations were performed on the potential energy surface (PES) of the H_2_-H_2_ system (*60*) within the Born-Oppenheimer (BO) approximation for the separation of electronic and nuclear motion. The PES is six-dimensional, i.e., it depends on the intermolecular distance, $\tilde{R}$, the three Jacobi angles, θ_1_, θ_2_, and φ = φ_1_ − φ_2_, and the intramolecular distances, *r*_1_ and *r*_2_ [see (*60*) for details]. Because the PES is calculated in the BO approximation, it can be used to study all possible combinations of hydrogen isotopologs, provided that the Jacobi angles are transformed accordingly (*61*–*63*). Here, we used the H_2_-H_2_ PES to study HD-HD collisions.

To solve the coupled channels equations, the PES was expanded over bispherical harmonics, $I_{l_{1}l_{2}l_{12}}\left(\theta_{1},\theta_{2},\varphi \right)$

$$V\left(\tilde{R},r_{1},r_{2},\theta_{1},\theta_{2},\varphi \right)=\sum_{l_{1}l_{2}l_{12}}A_{l_{1}l_{2}l_{12}}\left(\tilde{R},r_{1},r_{2}\right)I_{l_{1}l_{2}l_{12}}\left(\theta_{1},\theta_{2},\varphi \right)$$ (11)

where the bispherical harmonics are defined as

$$I_{l_{1}l_{2}l_{12}}\left(\theta_{1},\theta_{2},\varphi \right)=\sqrt{\frac{2l_{12}+1}{4\pi }}\sum_{m}C_{m,-m,0}^{l_{1}l_{2}l_{12}}\;Y_{l_{1}m}\left(\theta_{1},\varphi_{1}\right)Y_{l_{2},-m}\left(\theta_{1},\varphi_{2}\right)$$ (12)

Here, $C_{m,-m,0}^{l_{1}l_{2}l_{12}}$ are the Clebsch-Gordan coefficients, and $Y_{\mathrm{lm}}\left(\theta ,\varphi \right)$ denotes the spherical harmonic. Because both collisional partners are heteronuclear, the indices cover both even and odd values, with the restriction that $|l_{1}-l_{2}|\leq l_{12}\leq l_{1}+l_{2}$, and that the sum *l*_1_ + *l*_2_ + *l*_12_ is an even integer. We note that the expansion terms with odd *l*_1_ and *l*_2_ (which are absent in the expansion of the PES for the H_2_-H_2_ system) emerge due to the coordinate transformation (the center-of-mass shift). The sum over *l*_1_, *l*_2_, *l*_12_ was terminated at the 140th term corresponding to 6, 6, 12, which ensured the reconstruction of the initial HD-HD PES with a relative root mean square error at the level of $1.2\times 10^{-2}\%.$

The expansion coefficients, $A_{l_{1}l_{2}l_{12}}\left(\tilde{R},r_{1},r_{2}\right)$, were averaged over the intramolecular coordinates, *r_1_* and *r*_2_, corresponding to the spectroscopically active and perturbing molecules, respectively. The spectroscopically active molecule may be in the ground (ν=0) or the second excited (ν=2) vibrational states, while the perturbing molecule is always in the ground vibrational state (justified for the experimental temperature of 296 K). Thus, the average was performed by integrating the radial coupling terms with the weight corresponding to the squared modulus of the isolated HD wave function in the ν=0, j=3 and ν=2, j=2 rovibrational states (for the average over *r*_1_) and ν=0, j (for the average over *r*_2_), where j corresponds to the rotational level of the perturbing HD molecule. The wave functions of isolated HD molecules were obtained by solving the rovibrational Schrödinger equation for the HD molecule with the potential energy curve from (*64*) using the discrete variable representation–finite basis representation method.

The close-coupling equations were solved in the body-fixed frame (*65*) using the renormalized Numerov’s algorithm for energies in the range $E_{\text{kin}}\in \langle 10,1500\rangle \;{\text{cm}}^{-1}$ with various steps, to describe the channel-opening effects accurately. At sufficiently large $\tilde{R}$, the log-derivative matrix was transformed to the space-fixed frame, where boundary conditions were imposed, allowing for the recovery of S-matrix elements. The convergence of the scattering S-matrix is ensured by a proper choice of the integration range, propagator step, the size of the rovibrational basis, and the number of partial waves contributing to each scattering event (*65*). Calculations were performed using the in-house quantum scattering code BIGOS (*66*, *67*). We treat HD molecules as distinguishable collisional partners. This assumption is based on the theoretical and experimental evidence suggesting that quantum interference effects in collisions involving vibrationally excited molecules are negligible at thermal energies where many partial waves contribute to the scattering process (*68*).

The scattering S-matrix elements were used to calculate the generalized spectroscopic cross sections, $\sigma_{\lambda }^{q}\left(v_{i},j_{i},v_{f},j_{f},j_{2};E_{\text{kin}}\right)$ (*65*, *69*, *70*). Here, *q* is the tensor rank of the spectral transition operator (for the electric dipole transition considered here, *q* = 1) that drives the transition from the $\nu_{i}=0$, $j_{i}=3$ to the $\nu_{f}=2$, $j_{f}=2$ state in HD. The symbol $j_{2}$ denotes the rotational quantum number of the perturbing molecule, and λ is the rank of the velocity tensor. For λ=0, the real and imaginary parts of σ correspond to the standard pressure broadening (PBXS) and shift (PSXS) cross sections, respectively. For λ=1, the generalized spectroscopic cross section corresponds to the complex Dicke cross section associated with motion narrowing.

**Fig. 6.**
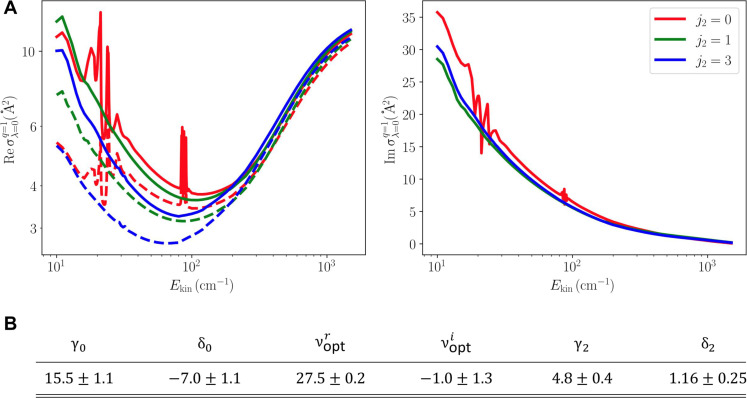
Ab initio line-shape parameters for the HD P(3) 2-0 transition. (**A**) Pressure broadening (left) and pressure shift (right) cross sections of the self-perturbed P(3) 2-0 line in HD, for selected rotational quantum numbers of the perturbing molecule. The dashed lines in the left panel correspond to the inelastic contribution to the pressure broadening cross section. (**B**) Table containing ab initio line-shape parameters (in 10^−3^ cm^−1^ atm^−1^), calculated for the HD P(3) 2-0 transition, used in the qSDHCP and SDBBP along with the estimated 1σ standard uncertainties.

Figure 6 presents the pressure broadening (left) and pressure shift (right) cross sections for the self-perturbed 2-0 band P(3) line in HD. The PBXS for all considered values of $j_{2}$ exhibits similar behavior to the one observed in He-perturbed lines of H_2_ (*71*) and HD (*72*). First, values of the cross sections decrease with increasing kinetic energy, then pass through a minimum in the vicinity of $E_{\text{kin}}\approx 100\;{\text{cm}}^{-1}$, and increase in the high-collision energy regime. The dashed lines in the top left of Fig. 6A present the inelastic contribution to collisional broadening, i.e., a half-sum of the total inelastic cross sections in the initial and final spectroscopic states [see equation 10 of (*73*)]. In contrast to He-perturbed HD lines (*70*), the inelastic contribution to the broadening of self-perturbed HD lines is much larger, constituting more than 90% of the broadening at kinetic energies larger than 100 cm^−1^. This is attributed to the presence of inelastic channels absent in atom-molecule collisions, i.e., rotational (de-)excitation of the perturber.

Both the PBXS and PSXS for *j*_2_ = 0 exhibit resonant features at kinetic energies in the vicinity of 23 and 90 cm^−1^. These features are associated with channel-opening effects: At $E_{\text{kin}}=22.8690\;{\text{cm}}^{-1}$, the $\left(\nu_{1},j_{1},\nu_{2},j_{2}\right)\to \left(\nu_{1}^{\prime },j_{1}^{\prime },\nu_{2}^{\prime },j_{2}^{\prime }\right)=\left(2,2,0,0\right)\to \left(2,0,0,2\right)$ process becomes energetically accessible. This represents a quasi-resonant transfer of rotational quanta ∆*j* = 2 between HD molecules, with an energy mismatch between the ladders of rotational levels in ν=0 and ν=3. The second resonant structure is related to the excitation of the perturbing molecule, while the active molecule remains in one of the spectroscopic states [the (0,3,0,0)→(0,3,0,1) and (2,2,0,0)→(2,2,0,1) transitions].

We averaged the $\sigma_{0}^{1}$ and $\sigma_{1}^{1}$ cross sections over the Maxwell-Boltzmann distribution of relative kinetic energies and summed the resulting values over the relative populations of HD to calculate the six line-shape parameters used in this work in qSDHCP and SDBBP:

• the speed-averaged collisional broadening and shift

$$\gamma_{0}-i\delta_{0}=\frac{1}{2\pi c}\frac{1}{k_{B}T}\langle v_{r}\rangle \sum_{j_{2}}p_{j_{2}}\left(T\right)\int_{0}^{\infty }\mathrm{dx}{\mathrm{xe}}^{-x}\sigma_{0}^{1}\left(j_{2};x\right)$$ (13)

• the speed dependences of collisional broadening and shift

$$\begin{array}{c} \gamma_{2}-i\delta_{2}=\frac{1}{2\pi c}\frac{1}{k_{B}T}\frac{\langle v_{r}\rangle \sqrt{M_{a}}}{2}e^{-y^{2}}\sum_{j_{2}}p_{j_{2}}\left(T\right)\\ \int_{0}^{\infty }d\bar{x}\left[2\bar{x}\text{cosh}\left(2\bar{x}y\right)-\left(\frac{1}{y}+2y\right)\text{sinh}\left(2\bar{x}y\right)\right]{\bar{x}}^{2}e^{-{\bar{x}}^{2}}\sigma_{0}^{1}\left(j_{2};\bar{x}\;{\bar{v}}_{p}\right) \end{array}$$ (14)

• the real and imaginary parts of the complex Dicke parameter

$$v_{\text{opt}}^{r}-iv_{\text{opt}}^{i}=\frac{1}{2\pi c}\frac{\langle v_{r}\rangle M_{a}}{k_{B}T}\sum_{j_{2}}p_{j_{2}}\left(T\right)\int_{0}^{\infty }\mathrm{dxx}e^{-x}\left[\frac{2}{3}x\sigma_{1}^{1}\left(j_{2};x\right)-\sigma_{0}^{1}(j_{2};x)\right]$$ (15)

Here, $\langle v_{r}\rangle =\sqrt{8k_{B}T/\pi \mu }$ is the mean relative speed of the colliding pair at a given temperature, ${\bar{v}}_{p}$ is the most probable speed of the perturbing molecule, *k*_B_ is the Boltzmann constant, μ is the reduced mass of the HD-HD system, and $M_{a}=m_{a}/\left(m_{a}+m_{p}\right)$, $x=E_{\text{kin}}/k_{B}T,$ $\bar{x}=v_{r}/{\bar{v}}_{p}$, $y=\sqrt{m_{p}/m_{a}}$, and *m*_a_ and *m*_p_ are the masses of the active and perturbing molecules, respectively. For the self-perturbed case considered here, the mass of the perturber (*m*_p_) and the active molecule (*m*_a_) is the same; hence, *M*_a_ = 1/2. Note that in $\sigma_{\lambda }^{1}$ symbols, we kept the quantum numbers related to the spectroscopically active molecule implicit for brevity. The quantity $p_{j_{2}}\left(T\right)$ is the population of the $j_{2}$ level at a given temperature

$$p_{j_{2}}\left(T\right)=\frac{\left(2j_{2}+1\right)e^{-E_{j_{2}}/k_{B}T}}{\sum_{j_{2}^{\prime }}\left(2j_{2}^{\prime }+1\right)e^{-E_{j_{2}^{\prime }}/k_{B}T}}$$ (16)

Summations over $j_{2}$ were truncated at $j_{2\text{max}}=3$, which covered 97% of the total population of the HD molecule at the experimental temperature. To account for the remaining 3% of the HD population at T=296 K, the cross sections were extrapolated for $j_{2}>3$ using values for $j_{2}=3$, justified by the lack of notable $j_{2}$ dependence of the cross sections, as seen in Fig. 6A. The final values of the line-shape parameters at 296 K are gathered in the table in Fig. 6B. Uncertainties of line-shape parameters were estimated following the procedure described in section 5 of (*71*).

### Statistical analysis

The intensity of the spectral line can be calculated as $S=A/N_{a}$, where 𝐴 is the line area determined from the line shape analysis and $N_{a}$ is the concentration of absorbers (number of molecules per volume) determined as a κ fraction of the total gas concentration N, $N_{a}=\kappa \;N$. Measuring the total pressure p of the gas sample and its temperature T, the total gas concentration N can be determined from the ideal gas law $N=p{\left(k_{B}T\right)}^{-1}$. As a result, we obtain the following formula for the line intensity S measured at temperature T, $S\left(T\right)=k_{B}\mathrm{TA}{\left(\kappa \;p\right)}^{-1}$. Note that the intensity of the line generally depends on the temperature. To take this into account, we define a temperature-dependent function $f_{S}\left(T\right)=\frac{S\left(T_{0}\right)}{S\left(T\right)}$, where $T_{0}$ is the reference temperature. The final expression for the line intensity determined for the reference temperature $T_{0}$ from a spectrum measured at temperature *T* is

$$S\left(T_{0}\right)=k_{B}f_{S}\left(T\right)\mathrm{TA}{\left(\kappa \;p\right)}^{-1}$$ (17)

The function $f_{S}\left(T\right)$ can be calculated based on the ratio of total internal partition functions provided by the HITRAN (high-resolution transmission molecular absorption) database (*45*).

To estimate the combined uncertainty u(S) of the line intensity determined for $T_{0}$, it should be noted that the quantities $f_{S}\left(T\right)$ and T in Eq. 17 are mutually dependent. Hence, the nonzero covariance of these quantities $C_{T,f_{s}}=\left(\frac{\partial f_{s}}{\partial T}\right)u^{2}\left(T\right)$ must be taken into account in the calculation of u(S). Assuming that the remaining quantities in Eq. 17 are independent of each other, we found the following formula for the square of the relative uncertainty of the line intensity determined for the temperature $T_{0}$

$$\begin{array}{c} {\left.\frac{u^{2}\left(S\right)}{S^{2}}\right|}_{T_{0}}=\left[\frac{1}{T^{2}}+\frac{1}{f_{s}^{2}}{\left(\frac{\partial f_{s}}{\partial T}\right)}^{2}+2\frac{1}{Tf_{s}}\frac{\partial f_{s}}{\partial T}\right]u^{2}\left(T\right)+\frac{u^{2}\left(p\right)}{p^{2}}+\\ \frac{u^{2}\left(A\right)}{A^{2}}+\frac{u^{2}\left(\kappa \right)}{\kappa^{2}} \end{array}$$ (18)

This expression was used to estimate the relative combined uncertainties of CO and HD line intensities reported here. The corresponding line intensity uncertainty budgets are presented in Tables 1 and 2. It should be noted that, when estimating the combined uncertainty of the CO line intensity, which is the average of the results of the dispersion HCRDS and CMDS methods, only the measurements of 𝐴 could be treated as independent, while the measurement of T, p, and κ was common to both methods. As a result, the combined uncertainty of the average line intensity u(〈S〉) was calculated by inserting the relative SD u(A)/A of the mean value of 𝐴 from both measurement methods into Eq. 18. Let us also note that, in the case of HD molecule, the quantity κ is determined not only by the isotope composition as it is for CO but also by the purity of the HD sample.

**Table 1.** Uncertainty budget for CO line intensity. This table contains the quantities *x* taken into account when estimating the combined CO line intensity uncertainty using Eq. 18. Type A standard uncertainties and type B standardized uncertainties of *x* and the corresponding combined uncertainties *u*(*x*) are given. Uncertainties of the line area, 𝐴, are shown for used measurement methods. The combined uncertainty of the line intensity *u*(*S*) is given for each method. We also show the value of *u*(〈*S*〉) estimated for the mean line intensity 〈*S*〉 obtained from the CMDS and HCRDS dispersion measurement results. For each measurement method, the bias of the line intensity from 〈*S*〉 is also shown. All values are given in per mil.

Method	Quantity, *x*	Type A (‰)	Type B (‰)	*u*(*x*)/*x*(‰)	*u*(*S*)/*S*(‰)	*u*(〈*S*〉)/〈*S*〉(‰)	Bias from 〈*S*〉 (‰)
	*T*	<0.01	0.1	0.1			
	*p*	0.05	0.62	0.62			
	κ		0.44	0.44			
CMDS	*A*	0.20	0.32	0.38	0.91	0.86	0.04
HCRDS (dispersion)	*A*	0.18	0.24	0.30	0.88		−0.04
CMWS	*A*	0.17	0.39	0.43	0.93		−0.34
HCRDS (absorption)	*A*	0.23	0.11	0.26	0.87		−0.75
CRDS	*A*	0.50	2.04	2.10	2.30		−9.34

**Table 2.** Uncertainty budget for HD line intensity. This table contains the quantities *x* taken into account when estimating the combined HD line intensity uncertainty using Eq. 17. Type A standard uncertainties and type B standardized uncertainties of *x* and the corresponding combined uncertainties *u*(*x*) are given. Uncertainties of the line area 𝐴 are shown for the various profiles that we used in line shape analysis. The combined uncertainty of the line intensity *u*(*S*) is given for each profile. For each profile, the bias of the line intensity from S*, obtained from SDBBP analysis with ab initio values of line-shape parameters, is also shown. All values are given in per mil.

Profile	Quantity, *x*	Type A (‰)	Type B (‰)	*u*(*x*)/*x*(‰)	*u*(*S*)/*S*(‰)	Bias from S* (‰)
	*T*	<0.01	0.1	0.1		
	*p*	0.05	0.62	0.62		
	κ		1	1		
SDNGP	*A*	1.29	0.93	1.59	1.98	−3.16
SDNGP (ab initio)	*A*	0.72	<0.01	0.72	1.38	−5.23
SDBBP	*A*	1.13	3.51	3.69	3.87	2.54
SDBBP * (ab initio)	*A*	0.36	<0.01	0.36	1.23	

It is worth mentioning how deviations from the ideal gas law affect the line intensity values reported here. We limit ourselves to the first correction in the expanded gas law $p=Nk_{B}T\left[1+B\left(T\right)N+C\left(T\right)N^{2}+\ldots \right]$, where B(T) and C(T) are virial coefficients of the second and third order, which reduces the expression for N to the form $N=p{\left(k_{B}T\right)}^{-1}\left[1-pp_{n}^{-1}T_{n}T^{-1}V_{n}^{-1}B\left(T\right)\right]$, where $p_{n}$, $T_{n}$, and $V_{n}$ describe normal conditions. For the measured CO and HD transitions, the virial coefficients B(T) determined at a temperature of 296 K are −8 cm^3^/mol (*74*) and +14 cm^3^/mol (*59*), respectively. They correspond to CO and HD line intensity relative corrections of −4.334 × 10^−6^ and 3.034 × 10^−5^, which were calculated for the highest pressures of 1.33 and 5.33 kPa, at which the spectra of CO and HD molecules were measured. These changes are at least two orders of magnitude lower than the accuracy of the determined here line intensities, so they are not included in the uncertainty budget.

### Sensitivity

**Fig. 7.**
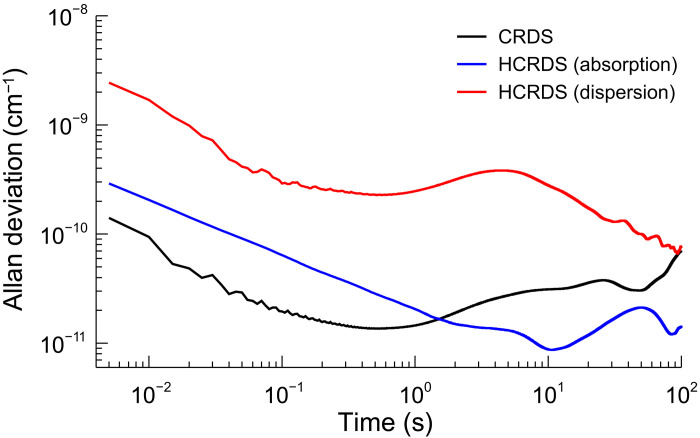
Sensitivity of spectroscopic measurements. Comparison of Allan deviation (AD) plots for measured mode positions (red) and widths (blue) in HCRDS method and ring-down decay time constants (black) in CRDS method. The AD was calculated on the basis of waveforms collected at a repetition rate of 200 Hz. Each 2.5-ms waveform recorded at a sampling frequency of 1.25 MHz was analyzed in a manner appropriate for a given spectroscopic method to obtain information on the absorption/dispersion of the sample. ADs of mode positions and widths originally expressed in frequency units were converted to absorption units (per centimeter).

The sensitivity of the HCRDS method was examined in terms of Allan variance analysis (*75*, *76*) and compared with sensitivity obtained with CRDS. To calculate the Allan deviation (AD) plots shown in Fig. 7, 40,000 waveforms corresponding to conventional and heterodyne ring-down decays were collected at repetition rate $f_{\text{rep}}=200$ Hz. Each 2.5 ms waveform recorded at a sampling frequency of 1.25 MHz was analyzed in a manner appropriate for a given spectroscopic method to obtain information on the absorption/dispersion of the sample. Measurements were performed for the cavity without an absorber and for a set of cavity mirrors with the geometric mean reflectance of 0.999993. The dataset volume was limited by the memory of the signal digitizer. ADs of CRDS time constants $\tau_{C,0}$ and $\sigma_{\tau }$ were converted to absorption units (per centimeter) using $\sigma_{\alpha }^{\text{CRDS}}=\sigma_{\tau }/c{\tau_{C,0}}^{2}$. For comparison with CRDS, the ADs of the width, $\sigma_{∆\gamma_{C}}$, and position, $\sigma_{∆\nu_{D}}$, of the cavity mode in HCRDS were converted to absorption units (per centimeter) using relations $\sigma_{\alpha }^{\text{HCRDS}\_\text{abs}}=4\pi c^{-1}\sigma_{∆\gamma_{C}}$ and $\sigma_{\alpha }^{\text{HCRDS}\_\text{disp}}=4\pi n_{0}c^{-1}{x\sigma }_{∆\nu_{D}}$, respectively, where $x=\text{Re}\left\{ℒ\left(\nu_{C}\right)\right\}/\text{Im}\left\{ℒ\left(\nu_{C}\right)\right\}$ for the frequency $\nu_{C}$ corresponding to the maximum resonant dispersion shift of the cavity mode. In the current calculations, x=0.68 refers to the ratio of the absorptive and dispersive HD spectrum taken on the same set of cavity mirrors as in the Allan variance measurements.

From the comparison of AD plots, it can be concluded that the absorptive HCRDS achieves sensitivity comparable to CRDS, but in 10 times longer time. The precision of cavity mode width measurements is additionally influenced by the frequency instability of the LO beam; hence, the AD plots for HCRDS start from a higher value. In the presented case, the minimum of AD plot for CRDS is 1.4 × 10^−11^ cm^−1^ and for absorptive HCRDS is 8.7 × 10^−12^ cm^−1^. Below this sensitivity, the slow drift and fluctuations of parasitic etalons typically occurring in spectroscopic systems begin to play a role. It should be noted that, to obtain a sufficiently high resolution of the Fourier spectrum, HCRDS needed four times longer time to register the light decay than CRDS under optimal conditions. This means that, under optimal conditions for CRDS, the slope of AD plot for CRDS should decrease twice as fast as for HCRDS, reaching lower AD values than HCRDS, provided that no signal fluctuations occur in the time domain.

**Fig. 8.**
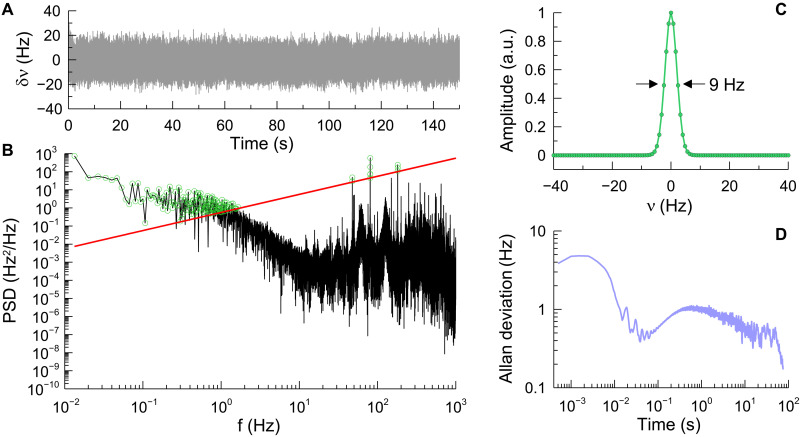
Laser frequency stability with respect to the optical cavity. (**A**) Transmission signal of the probe beam, detuned by 3 GHz from the locking point, recorded with sampling rate of 2 kHz on the mode slope of the cavity without the absorber. The signal amplitude is expressed in frequency units using a conversion factor of 1.43 mV/Hz derived from the cavity mode slope. (**B**) Power spectral density (PSD) of the signal from (A) (black) drawn together with the β-separation line (red) and marked PSD points (green) substantially contributing to the laser linewidth determined relative to the cavity mode. (**C**) Reconstructed laser line shape, determined relative to the cavity mode on the basis of the Fourier transform of an autocorrelation function using the PSD plot from (B). The derived relative laser linewidth is 9 Hz for an observation time of 1 ms. a.u., arbitrary units. (**D**) The AD plot of the signal from (A). Time-dependent instabilities at the laser-to-cavity locking point visible at the hertz level for times > 0.1s are caused by an uncompensated residual amplitude modulation of the PDH error signal.

The AD plot for dispersive HCRDS starts with the highest AD value in the comparison shown in Fig. 7. This is because the precision of cavity mode position measurements is influenced even more by the LO beam frequency noise than in cavity mode width measurements. Moreover, a large influence of the long-term frequency stability of the LO beam can be noticed here. The bump visible in the dispersive AD plot after 1 s of averaging is the result of fluctuations at the level of 1.2 Hz of the laser-to-cavity locking point (see Fig. 8D) caused by the residual amplitude modulation in the EOM of the PDH system (*48*). In the case of the presented dataset, a clear minimum of the AD was not obtained. The AD graph goes down to the value 6.6 × 10^−11^ cm^−1^, which we treat as the sensitivity of dispersion measurements in the current state of the HCRDS system. This value is in good agreement with our previous reports on sensitivity of dispersive measurements in the optical cavity [see figure 1b of (*21*)].

To provide information about the precision of the PDH relative locking of the laser to the cavity mode center, in Fig. 8B, we present a power spectral density (PSD) plot of the residual frequency noise of the locked laser measured using the slope of the cavity mode spectrum. The corresponding waveform is shown in Fig. 8A. By calculating the Fourier transform of the autocorrelation function using the measured PSD plot, the shape of the laser line can be reconstructed (*77*); see Fig. 8C. Direct integration of all regions where the PSD is larger than the β-separation line $8\text{ln}\left(2\right)f/\pi^{2}$, marked in green in Fig. 8B, allows the calculation of the approximate laser linewidth (*78*). The resulting laser linewidth relative to the cavity mode is 9 Hz with an observation time of 1 ms.

The calculated noise equivalent absorption coefficients, $\text{NEA}=\sigma_{\alpha }/\sqrt{f_{\text{rep}}}$, for CRDS, absorptive HCRDS, and dispersive HCRDS are 1.0 × 10^−11^ cm^−1^ Hz^−1/2^, 2.1 × 10^−11^ cm^−1^ Hz^−1/2^, and 1.7 × 10^−10^ cm^−1^ Hz^−1/2^, respectively. To check how far are these results from the quantum noise level, we used $\sigma_{\alpha }^{\text{shot}}=\sqrt{\tau_{C,0}\mathrm{eGT}/V_{0}}$ to calculate NEA^shot^ for CRDS, where e is an elementary charge, G is the detector gain, T is the transimpedance gain in volts per ampere, and $V_{0}$ is the measured signal voltage amplitude (*79*). For simulations of heterodyne ring-down signals (see the “Simulations” section), we added $\sigma_{V}^{\text{shot}}=\sqrt{2\text{eBGT}V_{0}}$, describing the shot-noise level voltage, where B is a bandwidth of the detector for the given G. In our case, *G* = 100, *T* = 627 V/A, and *B* = 8 MHz. The obtained NEA^shot^ values are 2.44 × 10^−13^ cm^−1^ Hz^−1/2^, 1.14 × 10^−13^ cm^−1^ Hz^−1/2^, and 1.34 × 10^−13^ cm^−1^ Hz^−1/2^, respectively, for CRDS, absorptive HCRDS, and dispersive HCRDS. Comparison of NEA and NEA^shot^ values shows that given sensitivities for CRDS and HCRDS methods are limited mainly by the technical noise of the detector and digitizer and additionally by the frequency stability of the LO beam in the case of HCRDS.

We estimated the dynamic range of the CRDS and HCRDS, defined as a ratio of the maximum to minimum measurable absorption coefficient or its dispersive equivalent obtained for our sets of cavity mirrors having *R* = 0.999925 and *R* = 0.999993. In HCRDS, a maximum of 1.6 × 10^−5^ cm^−1^ was achieved under the condition of not losing the per-mil precision of the measurement. Under the same condition for CRDS, the maximum was 1.0 × 10^−5^ cm^−1^, which is consistent with our previous measurements (*21*). These limits lead to dynamic ranges of 7.1 × 10^5^, 1.8 × 10^6^, and 2.4 × 10^5^, for CRDS, absorptive HCRDS, and dispersive HCRDS, respectively.
